# Epidemiology of Serotype 1 Invasive Pneumococcal Disease, South Africa, 2003–2013

**DOI:** 10.3201/eid2202.150967

**Published:** 2016-02

**Authors:** Claire von Mollendorf, Cheryl Cohen, Stefano Tempia, Susan Meiring, Linda de Gouveia, Vanessa Quan, Sarona Lengana, Alan Karstaedt, Halima Dawood, Sharona Seetharam, Ruth Lekalakala, Shabir A. Madhi, Keith P. Klugman, Anne von Gottberg, for the Group for Enteric, Respiratory, and Meningeal Disease Surveillance in South Africa (GERMS-SA)

**Affiliations:** National Institute for Communicable Diseases, Johannesburg, South Africa (C. von Mollendorf, C. Cohen, S. Tempia, S. Meiring, L. de Gouveia, V. Quan, S. Lengana, S.A. Madhi, K.P. Klugman, A. von Gottberg);; University of the Witwatersrand, Johannesburg (C. von Mollendorf, C. Cohen, A. Karstaedt, S. Seetharam, S.A. Madhi, A. von Gottberg);; Centers for Disease Control and Prevention, Atlanta, Georgia, USA (S. Tempia), and Pretoria, South Africa (S. Tempia);; Chris Hani Baragwanath Academic Hospital, Johannesburg (A. Karstaedt, S. Seetharam);; Pietermaritzburg Metropolitan Hospital, Pietermaritzburg, South Africa (H. Dawood);; University of KwaZulu-Natal, Pietermaritzburg (H. Dawood);; National Health Laboratory Service, Johannesburg (S. Seetharam);; National Health Laboratory Service, Polokwane, South Africa (R. Lekalakala);; University of Limpopo, Polokwane (R. Lekalakala);; Emory University, Atlanta, Georgia, USA (K.P. Klugman)

**Keywords:** Streptococcus pneumoniae, serotype 1, South Africa, pneumococcal conjugate vaccine, PCV13, epidemiology, surveillance, invasive, vaccination, incidence, pneumonia, bacteremia, immunization, streptococci

## Abstract

In South Africa, 7-valent pneumococcal conjugate vaccine (PCV) was introduced in April 2009 and replaced with 13-valent PCV in April 2011. We describe the epidemiology of serotype 1 *Streptococcus pneumoniae* disease during the pre- and post-PCV eras (2003–2013). Using laboratory-based invasive pneumococcal disease (IPD) surveillance, we calculated annual incidences, identified IPD clusters, and determined serotype 1–associated factors. Of 46,483 IPD cases, 4,544 (10%) were caused by serotype 1. Two clusters of serotype 1 infection were detected during 2003–2004 and 2008–2012, but incidence decreased after 2011. Among children <5 years of age, those who had non–serotype 1 IPD had shorter hospital stays, fewer cases of penicillin-nonsusceptible disease, and lower HIV prevalence and in-hospital death rates than did those with serotype 1 IPD; similar factors were noted for older patients. Serotype 1 IPD had distinctive clinical features in South Africa, and annual incidences fluctuated, with decreases noted after the introduction of PCV13.

## Abstract 2

Because of the epidemic nature of this disease and its distinctive clinical features in this area, surveillance should continue.

*Streptococcus pneumonia*e serotype 1 is highly invasive and rarely carried asymptomatically (*1*). The incidence of serotype 1 invasive pneumococcal disease (IPD) fluctuates year to year; disease is associated with outbreaks in closed communities and hospitals and, in Africa, with communitywide meningitis outbreaks (*2*). Compared with other *S. pneumonia*e serotypes, serotype 1 tends to cause fewer cases of fatal disease, and antibiotic-resistant cases are unusual (*1*).

IPD is common in children with underlying diseases, especially HIV. A study conducted among children <18 years of age in Israel before introduction of 7-valent pneumococcal conjugate vaccine (PCV7) showed that, compared with other common serotypes, serotype 1 caused more bacteremic pneumonia and peritonitis, occurred in older children and certain ethnic groups, and affected otherwise healthy children (*3*). After PCV7 introduction, infections caused by serotypes included in the vaccine declined, but other pneumococcal serotypes (e.g., serotype 1, which was later included in 13-valent vaccine [PCV13]) became relatively more common (*4*–*6*); serotype 1 ranked among the top 4 serotypes infecting children <5 years of age (*7*). Although PCV7 use may have contributed to the relative increase in serotype 1 infections, some studies showed no correlation between the vaccine and serotype 1 disease incidence (*8*). Lack of correlation is likely due to the epidemic-prone nature of serotype 1 disease and annual fluctuations in disease incidence (*9*). In addition, replacement disease is mainly due to common colonizing serotypes. An indirect cohort analysis using data from the United Kingdom Health Protection Agency (now Public Health England) surveillance program could not demonstrate significant protection against serotype 1 IPD by PCV13, although the point estimate suggested protection (vaccine effectiveness 62% [95% CI −112% to 92%]) (*10*). Two trials of a 9-valent vaccine showed waning protection against serotype 1 in the absence of a booster vaccine dose in the second year of life; vaccine failures clustered in children >18 months of age (*11*,*12*).

In South Africa, PCV7 was introduced into the national immunization schedule in April 2009 as a 3-dose regimen for infants 6 weeks, 14 weeks, and 9 months of age; in April 2011, the vaccine was replaced with PCV13. Among children <1 year of age, reported coverage for the third dose of PCV improved from 10% in 2009 to 81% in 2012 but declined to 62% in 2013 (*13*). In 2012, after PCV13 introduction, serotype 1 IPD incidence showed a temporally associated decline in children <2 years of age (−57%, 95% CI −79% to −16%) and adults 25–44 years of age (−33%, 95% CI −46% to −17%) compared with incidence in 2005–2008 (*14*).

Information regarding *S. pneumonia*e serotype 1 epidemiology in Africa is limited. We compared serotype 1 disease epidemiology in South Africa with that of other serotypes over an 11-year period, before and after introduction of PCV7 and PCV13. We also explored whether temporal or spatial clusters of serotype 1 disease occurred during the study period.

## Methods

### Study Design and Setting

Persons of any age were included in the study if they were hospitalized in South Africa during 2003–2013 for laboratory-confirmed IPD and had an available *S. pneumoniae* serotype result for an isolate from a normally sterile site. Patients were identified through an active national, laboratory-based surveillance program for *S. pneumoniae*. Data were contributed by >200 hospital-based diagnostic laboratories that submitted pneumococcal isolates to the National Institute for Communicable Diseases, Johannesburg, South Africa. Most laboratories were nonenhanced sites where only isolates and accompanying laboratory report forms with patient age, sex, date and source of the specimen were submitted. However, 24 sites (primarily tertiary hospitals) implemented enhanced surveillance, in which dedicated surveillance officers collected additional clinical information on identified patients; at least 1 site was located in each South Africa province, giving national representation (*14*). Enhanced sites were chosen on the basis of convenience, interest from site investigators, and number of isolates submitted each year; thus, some differences existed between enhanced and nonenhanced sites (Technical Appendix). Annual audits conducted by using a laboratory-based information system were used to identify unreported cases, which were included and used in incidence calculations.

Participants identified from enhanced and nonenhanced sites were included for determining incidence rates and cluster mapping. For the analyses of factors associated with serotype 1 pneumococcal disease and fatality, only participants from enhanced sites with detailed clinical information and known in-hospital outcomes were included.

Approval was obtained from the Human Research Ethics Committee (Medical), University of the Witwatersrand, Johannesburg (M081117), and other hospital or provincial ethics committees, as required. Informed consent was obtained for all patients.

### Definitions

IPD cases were defined as disease in persons with *S. pneumoniae* detected in cultures of specimens from normally sterile sites or persons with culture-negative samples that were positive by latex agglutination and/or Gram stain microscopy or *lytA* PCR (*15*). Pneumococci were serotyped by the Quellung method (Statens Serum Institut, Copenhagen, Denmark).

Serotype 1 clusters were defined as an increase in serotype 1 IPD numbers relative to other serotype numbers in a specific geographic area and time. Cluster location was based on hospital district where cases were diagnosed; actual geographic location was considered to be the centroid of the district polygon. Other definitions are provided in the online Technical Appendix.

### Incidence Estimations

We calculated annual incidence of serotype 1 disease per 100,000 population during 2003–2013 by using data for participants in defined age groups. We divided the number of age-specific, culture-positive serotype 1 IPD cases reported each year by age-specific midyear population estimates. Incidences for non–serotype 1 disease were similarly calculated. Serotype data for cases without serotype results from culture (including cases with only PCR serotype results) were imputed by age and year to obtain final incidence rates. Missing data were assumed to be random among different serotypes. Midyear population denominators were obtained from Statistics South Africa (http://www.statssa.gov.za/). To show differences in serotype incidences between prevaccine and postvaccine years, we compared an average incidence from prevaccine years (2003–2008) to 1 postvaccine year (2013). As a baseline for comparison, we included the average for years without clusters (2005–2007). CIs were calculated by using Poisson distribution for incidence rates.

### Factors Associated with Serotype 1 IPD and Case-Fatality Rates

For the analyses of factors associated with serotype 1 IPD, we included only participants with culture- and PCR-positive results from enhanced sites during 2003–2013. Patients were stratified into 2 age groups (<5 and >5 years), and disease-associated factors in those with serotype 1 IPD were compared with those in patients with non–serotype 1 IPD by using a multivariable logistic regression model. A second model to assess in-hospital fatalities restricted the analysis to serotype 1 IPD cases.

For both models, we assessed all variables considered significant (p<0.2) on univariate analysis and removed nonsignificant factors (p>0.05) by manual backward elimination. Patients with missing data for included variables were excluded. Statistical analysis was implemented by using Stata version 13.1 (StataCorp LP, College Station, TX, USA).

### Spatiotemporal Analysis for Detection of Serotype 1 IPD Clusters

We conducted a space–time scan analysis to detect serotype 1 clusters by aggregating IPD cases with available serotype results from January 2003–December 2013 by month and district. To minimize potential biases introduced by temporal and geographic differences in specimen-collecting practices, healthcare-seeking behavior, or surveillance system improvements over time, we compared cases (serotype 1 IPD cases) with controls (non–serotype 1 IPD cases) from the same geographic area and time period; a Bernoulli model (*16*,*17*) was used for the comparison.

To account for control number reductions after PCV7 introduction, we adjusted (increased) observed control numbers by the percent reduction from the prevaccine period (*14*). To obtain estimated monthly numbers of controls, assuming no PCV introduction, we linearly interpolated estimated annual proportional reductions from June to June of consecutive years from 2009 through 2013. Because the percentage of reduction in the control numbers may have differed by geographic area due to locality differences in PCV7 uptake over time, we obtained monthly adjustment factors for each province. This adjustment would decrease the likelihood of detecting a cluster if, in fact, a cluster did not occur (null hypothesis).

To identify spatial clusters, we used an elliptical area of search that was allowed to vary in size, shape, and direction. Significance was assessed at p<0.05 over 999 replications. Space-time analysis was conducted by using SaTScan version 9.3.1 (http://www.satscan.org/); maps were generated by using ArcGIS version 9.2 (http://www.esri.com/). To calculate relative risks for districts, we divided observed number of cases by expected number of cases in each district.

## Results

**Figure 1:**
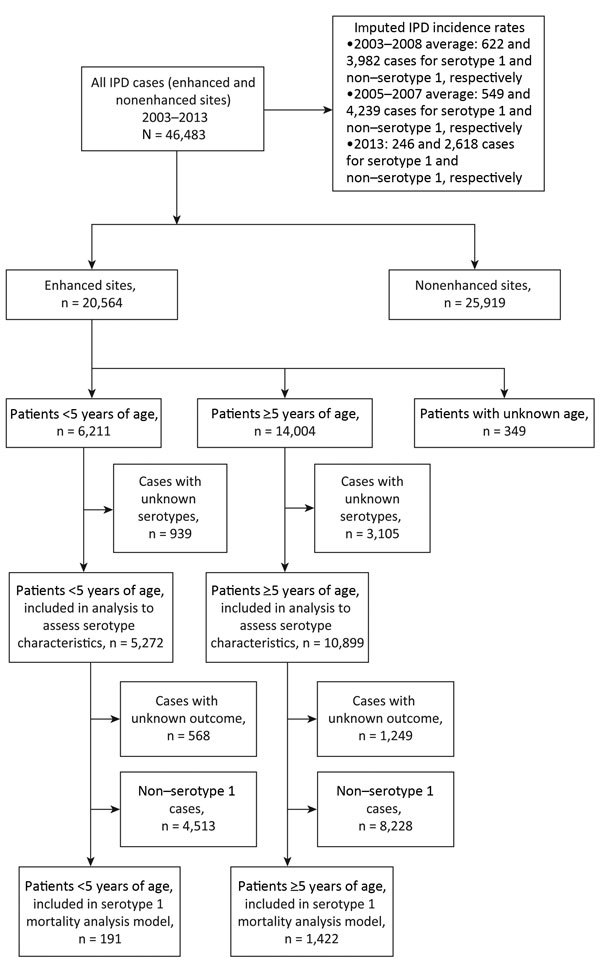
Selection flowchart for study of invasive *Streptococcus pneumoniae* disease (IPD) cases in South Africa, 2003–2013. Cases were reported by Group for Enteric Respiratory and Meningeal Disease Surveillance sites (GERMS-SA). Years indicate prevaccine (2003–2008), baseline (2005–2007), and postvaccine (2013) periods. Nonenhanced sites only submitted isolates and accompanying laboratory report forms, which included patient age and sex and the date and source of the specimen; enhanced sites (primarily tertiary hospitals) implemented enhanced surveillance, in which dedicated surveillance officers collected additional clinical information on identified patients.

During 2003–2013, a total of 46,483 persons with IPD were enrolled in the study; 32,841 (71%) had viable isolates and known *S. pneumoniae* serotype, and 1,204 (3%) had serotype determination by PCR. Of the 46,483 persons, 20,564 (44%) were enrolled from enhanced sites; of these 6,211 (30%) were <5 years of age, 14,004 (68%) were >5 years of age, and 349 (2%) had unknown age (Figure 1). Of the 4,985 patients who died, 68% (3,365) did so within 3 days of admission. Of the 12,013 patients who recovered, 14% (1,673) were hospitalized for <3 days, 62% (7,427) for 4–14 days, and 24% (2,913) for >2 weeks. In the pre-PCV7 period (2003–2008), serotype 1 was the sixth most common *S. pneumoniae* serotype among children <5 years of age, but by 2013, it was eleventh. In contrast, among persons >5 years of age, serotype 1 was the most common serotype across all years, although case numbers decreased after PCV13 introduction.

### Comparison of Enhanced and Nonenhanced Sites

Characteristics of enhanced and nonenhanced sites differed (Technical Appendix Table 1). Compared with nonenhanced sites, enhanced sites had a higher proportion of cases among younger children, more cases from certain provinces, fewer cases in 2012–2013, more penicillin-nonsusceptible cases, more blood culture results, and fewer serotype 1 IPD cases.

### Incidence of Serotype-Specific IPD in Different Age Groups

**Figure 2:**
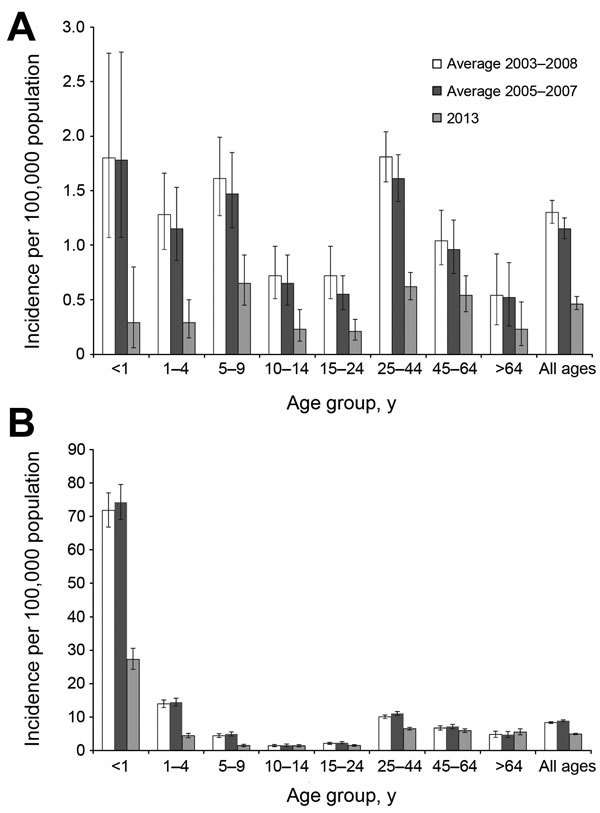
Incidence of serotype 1 and non–serotype 1 invasive pneumococcal disease (IPD) by age group, South Africa, 2003–2013. Years indicate prevaccine (2003–2008), baseline without clusters (2005–2007), and postvaccine (2013) periods. A) Serotype 1 IPD incidence by age group during prevaccine (no. cases = 622), baseline (no. cases = 549), and postvaccine (no. cases = 246) years. B) Non–serotype 1 IPD incidence by age group during prevaccine (no. cases = 3,982), baseline (no. cases = 4,239), and postvaccine years (no. cases = 2,618). Error bars indicate 95% CIs.

During the prevaccine era (2003–2008), serotype 1 incidence per 100,000 population was highest among persons <1 (1.8 cases), 5–9 (1.6 cases), and 25–44 (1.8 cases) years of age (Figure 2, panel A). Serotype 1 incidence did not differ significantly for 2003–2008 compared with 2005–2007, when there were no clusters. In 2013, serotype 1 incidence was highest among persons 5–9 (0.7 cases) and 25–44 (0.6 cases) years of age; reductions were significant (p<0.001) in all age groups except the >64-year-old age group (p = 0.07).

For all other serotypes during 2003–2008, the highest incidence rates per 100,000 population were among persons <1 (71.8 cases), 1–4 (13.9 cases), and 25–44 (10.1 cases) years of age (Figure 2, panel B). In 2013, the highest incidence rates were among persons <1 (27.3 cases) and >25 (>5.0 cases) years of age. Reductions in incidence among persons <5 and 25–44 years of age were significant (p<0.001).

The incidence of serotype 1 IPD fluctuated over the 11-year period (Technical Appendix Figure 1). For the <5-year-old age group, incidence rates were significantly reduced in 2006 (p = 0.01), 2007 (p = 0.03), 2010 (p = 0.006), and 2012–2013 (p<0.001) compared with rates in 2005. In the >5-year-old age group, incidence rates were significantly higher in 2003 (p = 0.001) and 2004 (p = 0.002) compared with 2005 but lower during 2006–2008 and 2010–2013 (p<0.001).

### Factors Associated with Serotype 1 IPD

After adjustment for geographic location (province), year (based on prominent serotype 1 fluctuations), and clinical syndrome, we saw a difference among patients at enhanced sites who had IPD caused by serotype 1 versus other serotypes. Multivariable analysis showed a difference in disease distribution by province, year, and age among children <5 years of age; these difference were more apparent in children >3 than <1 years of age. Compared with children with non–serotype 1 IPD, those with serotype 1 disease had significantly shorter hospitalizations (<3 days vs. 4–14 days [odds ratio (OR) 0.58, 95% CI 0.33–1.02] or >15 days [OR 0.44, 95% CI 0.23–0.85]) and were less likely to have HIV disease (OR 0.19, 95% CI 0.12–0.31), to die while hospitalized (OR 0.38, 95% CI 0.19–0.76), or to have penicillin-nonsusceptible disease (OR 0.02, 95 CI 0.01–0.05) (Table 1).

**Table 1:** Characteristics of 5,272 patients <5 years of age with invasive pneumococcal disease caused by serotype 1 or non–serotype 1 *Streptococcus pneumoniae*, South Africa, 2003–2013*

Variable	No. cases/no. total (%)			Univariate analysis†			Multivariable analysis†	
	Serotype 1	Non–serotype 1		OR (95% CI)	p value		aOR (95% CI)	p value
Age, y								
<1	63/211 (30)	2,754/5,061 (54)		Reference	<0.001		Reference	<0.001
1	35/211 (17)	1,155/5,061 (23)		1.32 (0.87–2.01)			2.36 (1.31–4.26)	
2	43/211 (20)	519/5,061 (10)		3.62 (2.43–5.40)			6.91 (3.78–12.64)	
3	37/211 (18)	355/5,061 (7)		4.56 (2.99–6.94)			12.03 (6.12–23.64)	
4	33/211 (16)	278/5,061 (5)		5.19 (3.35–8.05)			7.13 (3.60–14.13)	
Province								
Gauteng	95/211 (45)	2,067/5,061 (41)		Reference	<0.001		Reference	<0.001
Western Cape	11/211 (5)	1,158/5,061 (23)		0.21 (0.11–0.39)			0.11 (0.04–0.26)	
KwaZulu-Natal	46/211 (22)	957/5,061 (19)		1.05 (0.73–1.50)			1.04 (0.59–1.84)	
Eastern Cape	15/211 (7)	152/5,061 (3)		2.15 (1.22–3.79)			1.98 (0.74–5.28)	
Free State	25/211 (12)	383/5,061 (8)		1.42 (0.90–2.24)			1.06 (0.56–2.00)	
Mpumalanga	4/211 (2)	104/5,061 (2)		0.84 (0.30–2.32)			0.58 (0.07–4.86)	
North-West	5/211 (2)	46/5,061 (1)		2.36 (0.92–6.09)			5.65 (1.33–24.05)	
Limpopo	4/211 (2)	48/5,061 (1)		1.81 (0.64–5.13)			1.79 (0.41–7.90)	
Northern Cape	6/211 (3)	146/5,061 (3)		0.89 (0.39–2.08)			0.50 (0.15–1.64)	
Year of specimen collection								
2003	31/211 (15)	544/5,061 (11)		1.20 (0.72–1.99)	0.004		1.10 (0.49–2.49)	0.05
2004	26/211 (12)	699/5,061 (14)		0.78 (0.46–1.32)			0.58 (0.25–1.34)	
2005	32/211 (15)	672/5,061 (13)		Reference			Reference	
2006	21/211 (10)	551/5,061 (11)		0.80 (0.46–1.40)			0.77 (0.34–1.72)	
2007	15/211 (7)	547/5,061 (11)		0.58 (0.31–1.07)			0.67 (0.26–1.75)	
2008	10/211 (5)	542/5,061 (11)		0.39 (0.19–0.80)			0.40 (0.15–1.03)	
2009	23/211 (11)	494/5,061 (10)		0.98 (0.57–1.69)			1.43 (0.63–3.24)	
2010	19/211 (9)	361/5,061 (7)		1.11 (0.62–1.98)			0.82 (0.33–2.08)	
2011	19/211 (9)	240/5,061 (5)		1.66 (0.92–2.99)			1.04 (0.44–2.44)	
2012	12/211 (6)	190/5,061 (4)		1.33 (0.67–2.63)			0.49 (0.18–1.33)	
2013	3/211 (1)	221/5,061 (4)		0.29 (0.09–0.94)			0.12 (0.02–0.59)	
Medical conditions/treatment								
Length of hospital stay, d								
<3	57/186 (31)	1,238/4,489 (28)		Reference	0.09		Reference	0.04
4–14	96/186 (52)	2,138/4,489 (48)		0.98 (0.70–1.36)			0.58 (0.33–1.02)	
>15	33/186 (18)	1,113/4,489 (25)		0.64 (0.42–1.00)			0.44 (0.23–0.85)	
Previously hospitalized	39/164 (24)	1,676/4,110 (41)		0.45 (0.31–0.65)	<0.001			
Underlying medical condition‡	27/114 (24)	1,321/3,371 (39)		0.48 (0.31–0.75)	0.001			
Antimicrobial drug use in previous 2 mo§	10/147 (7)	742/3,549 (21)		0.28 (0.14–0.53)	<0.001			
HIV infected	43/132 (33)	2,125/3,539 (60)		0.32 (0.22–0.47)	<0.001		0.19 (0.12–0.31)	<0.001
TB treatment in previous 3 mo	11/161 (7)	570/3,928 (15)		0.43 (0.23–0.80)	0.008			
Malnourished¶	24/95 (25)	1,109/2,619 (42)		0.46 (0.29–0.74)	0.001			
Died during hospitalization	24/191 (13)	1,105/4,513 (24)		0.44 (0.29–0.68)	<0.001		0.38 (0.19–0.76)	0.006
Pneumococcal isolate characteristics								
Penicillin nonsusceptible#	4/203 (2)	2,580/4,950 (52)		0.02 (0.01–0.05)	<0.001		0.02 (0.01–0.05)	<0.001
Previous invasive pneumococcal disease**	2/211 (1)	356/5,061 (7)		0.13 (0.03–0.51)	0.004			
Clinical syndrome††								
Meningitis	59/198 (30)	1,668/4,736 (35)		Reference	0.001			
Pneumonia	124/198 (63)	2,358/4,736 (50)		1.49 (1.08–2.04)				
Bacteremia	15/198 (8)	710/4,736 (15)		0.60 (0.34–1.06)				

*All patients were reported from the enhanced Group for Enteric, Respiratory, and Meningeal Disease Surveillance in South Africa (GERMS-SA) surveillance sites. aOR, adjusted odds ratio; OR, odds ratio; TB, tuberculosis. †Only variables significant on univariate and multivariable analysis are shown. Variables not included are sex, race, Pitt bacteremia score, prematurity, antimicrobial drug use in previous 24 h, viable culture, and specimen type. ‡Includes asplenia or sickle cell anemia; chronic illness (i.e., chronic lung, renal, liver, cardiac disease, and diabetes); other immunocompromising conditions (i.e. including organ transplant, primary immunodeficiency, immunotherapy, and malignancy, but excluding HIV); and other risk factors (i.e., head injury with possible cerebral spinal fluid leak, neurologic disorders, burns, and chromosomal abnormalities). Excludes malnutrition. §Use of any antimicrobial drug in 2 mo prior to admission. ¶Malnutrition was classified as a weight-for-age *z*-score of less than −2 (World Health Organization child growth standards 2009) (*18*), nutritional edema, or both. #Considered penicillin nonsusceptible at MIC >0.12 μg/mL; intermediately resistant and resistant groups were combined into a nonsusceptible group. **Invasive pneumococcal disease diagnosis >21 d before this episode. ††Clinical diagnoses were made on the basis of documented discharge diagnoses in patient medical records; clinical syndrome were separated into 3 groups: meningitis, bacteremic pneumonia, and bacteremia without focus or other diagnosis (e.g., septic arthritis, endopthalmitis, peritonitis, pericarditis).

Among persons >5 years of age, serotype 1 IPD (compared with non–serotype 1 IPD) was significantly associated with province, year, and patient age: compared with persons >64 years of age, ORs (95% CIs) were 13.48 (5.53–32.82) for children 5–9 years of age; 8.02 (3.15–20.43) for children 10–14 years of age; 5.65 (2.31–13.82) for persons 15–24 years of age; 3.67 (1.53–8.76) for persons 25–44 years of age; and 2.57 (1.06–6.23) for persons 45–64 years of age (Technical Appendix Table 2). Compared with persons with non–serotype 1 IPD, those with serotype 1 disease had significantly shorter hospitalization (<3 days vs. 4–14 days [OR 0.86, 95% CI 0.68–1.09] and vs. >15 days [OR 0.64, 95% CI 0.48–0.86]) and lower rates of previous admissions (OR 0.45, 95% CI 0.35–0.57) and tuberculosis treatment (OR 0.73, 95% CI 0.57–0.95).

Persons >5 years of age with serotype 1 disease were also significantly less likely to have HIV (OR 0.39, 95% CI 0.31–0.49) or penicillin-nonsusceptible disease (OR 0.02, 95% CI 0.01–0.04), and they were more likely than those with non–serotype 1 IPD to receive a diagnosis of pneumonia (OR 1.28, 95% CI 1.03–1.58) or bacteremia (OR 1.76, 95% CI 1.22–2.55) rather than meningitis. In-hospital death compared with recovery was not significant in the >5 year age group.

### Factors Associated with In-Hospital Deaths among Patients with Serotype 1 IPD

We conducted multivariable analysis to explore factors associated with death in children <5 years of age with serotype 1 IPD (Table 2). Compared with 4-year-old children, those <1 year of age were more likely to die (OR 12.06, 95% CI 1.45–100.26), as were children with underlying medical conditions than those without. Odds of death were also increased among children with HIV (OR 2.82, 95% CI 1.36–5.84) or meningitis versus those with pneumonia or bacteremia. Duration of hospitalization was shorter among persons who died compared with those who recovered (<3 days vs. 4–14 days [OR 0.06, 95% CI 0.03–0.15] or >15 days [OR 0.02, 95% CI 0.01–0.07]).

**Table 2:** Factors associated with death in patients <5 years of age with serotype 1 invasive pneumococcal disease, South Africa, 2003–2013*

Variable	Univariate analysis				Multivariable analysis	
	No. deaths/no. cases (%)	OR (95% CI)	p value		aOR (95% CI)	p value
Age group, y						
<1	102/355 (29)	11.49 (2.75–47.95)	<0.001		12.06 (1.45–100.26)	0.02
1	22/154 (14)	4.75 (1.08–20.88)			3.83 (0.41–35.35)	
2	11/94 (12)	3.78 (0.81–17.69)			1.30 (0.12–14.34)	
3	6/73 (8)	2.55 (0.49–13.14)			1.40 (0.12–15.82)	
4	2/59 (3)	Reference			Reference	
Province						
Gauteng	53/327 (16)	Reference	0.001			
Western Cape	15/111 (14)	0.81 (0.44–1.50)				
KwaZulu-Natal	26/111 (23)	1.58 (0.93–2.68)				
Eastern Cape	12/44 (27)	1.94 (0.94–4.01)				
Free State	11/62 (18)	1.11 (0.55–2.28)				
Mpumalanga	7/19 (37)	3.02 (1.13–8.01)				
North-West	11/23 (48)	4.74 (1.99–11.30)				
Limpopo	7/21 (33)	2.58 (1.00–6.71)				
Northern Cape	1/17 (6)	0.32 (0.04–2.49)				
Medical condition/treatment						
Length of hospital stay, d						
<3	94/209 (45)	Reference	<0.001		Reference	<0.001
4–14	36/354 (10)	0.14 (0.09–0.21)			0.06 (0.03–0.15)	
>15	10/160 (6)	0.08 (0.04–0.16)			0.02 (0.01–0.07)	
Pitt bacteremia score†						
0–3	102/608 (17)	Reference	<0.001			
>4	16/28 (58)	6.61 (3.04–14.40)				
Underlying medical condition‡						
No	55/343 (16)	Reference	0.19		Reference	0.003
Yes	33/158 (21)	1.38 (0.86–2.23)			3.21 (1.49–6.91)	
Antimicrobial drug use in 24 h before admission						
No	82/504 (16)	Reference	0.05			
Yes	15/56 (26)	1.88 (1.00–3.56)				
HIV status						
HIV-uninfected	37/252 (15)	Reference	0.13		Reference	0.005
HIV-infected	52/263 (20)	1.43 (0.90–2.27)			2.82 (1.36–5.84)	
Malnourished§						
No	44/277 (16)	Reference	0.03			
Yes	43/176 (24)	1.71 (1.07–2.74)				
Clinical syndrome/specimen type						
Specimen type						
CSF	59/166 (36)	Reference	<0.001			
Blood	83/530 (16)	0.34 (0.23–0.50)				
Other	1/39 (3)	0.05 (0.01–0.36)				
Clinical syndrome¶						
Meningitis	74/209 (35)	Reference	<0.001		Reference	0.0003
Pneumonia	50/410 (12)	0.25 (0.17–0.38)			0.25 (0.11–0.54)	
Bacteremia	18/111 (16)	0.35 (0.20–0.63)			0.11 (0.03–0.42)	

*All patients were reported from the enhanced Group for Enteric, Respiratory, and Meningeal Disease Surveillance in South Africa (GERMS-SA) surveillance sites. Only variables significant on univariate and multivariable analysis are shown. Variables not included in table are sex, year, previous hospital admission, prematurity, antimicrobial drug in previous 2 mo, and penicillin nonsusceptible invasive pneumococcal disease. aOR, adjusted odds ratio; OR, odds ratio. †Pitt bacteremia score calculated using temperature, hypotension, mechanical ventilation, cardiac arrest and mental status. Severe disease defined as score of >4 points. ‡Includes asplenia or sickle cell anemia; chronic illness (i.e., chronic lung, renal, liver, cardiac disease, and diabetes); other immunocompromising conditions (i.e., organ transplant, primary immunodeficiency, immunotherapy, and malignancy, but excluding HIV); and other risk factors (i.e., head injury with possible cerebral spinal fluid leak, neurologic disorders, burns, and chromosomal abnormalities). Excludes malnutrition. §Children with weight-for-age *z*-score of less than −2 (World Health Organization child growth standards 2009) (*18*), nutritional edema, or both. ¶Clinical diagnoses were made on the basis of documented discharge diagnoses in patient medical records, with clinical syndrome separated into 3 groups: meningitis, bacteremic pneumonia, and bacteremia without focus or other diagnosis (e.g., septic arthritis, endopthalmitis, peritonitis, pericarditis)

Similar factors were associated with increased odds of death in persons >5 years of age with serotype 1 IPD (Technical Appendix Table 3). In addition, death was more likely among persons who had received tuberculosis treatment in the previous 3 months (OR 1.75, 95% CI 1.25–2.45) and among severely ill persons (OR 5.26, 95% CI 3.53–7.84 for patients with a Pitt bacteremia score >4). No difference was seen in the odds of death by HIV status. Compared with children 5–9 years of age, persons >25 years of age had incrementally increased odds of death by age group: 25–44 years of age, OR 5.07 (95% CI 2.74–9.38); 45–64 years of age, OR 9.00 (95% CI 4.66–17.35); and >64 years of age, OR 10.13 (95% CI 4.46–23.00).

### Detection of Serotype 1 IPD Clusters

**Figure 3:**
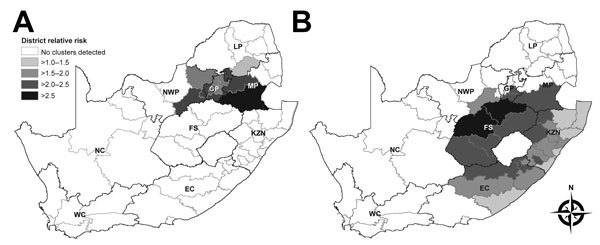
Serotype 1 invasive pneumococcal disease clusters by district, South Africa. A) May 2003–December 2004. B) September 2008–April 2012. Gray borders indicate district boundaries; black borders indicate provincial boundaries. Provinces: EC, Eastern Cape; FS, Free State; GP, Gauteng; KZN, KwaZulu-Natal; LP, Limpopo; MP, Mpumalanga; NC, Northern Cape; NWP, North-West; WC, Western Cape. District relative risk was calculated by dividing the observed number of cases per district by the number of cases expected by district (as determined on the basis of numbers in control groups).

Of the 46,483 IPD cases, 34,032 (73%) had available data (i.e., date of specimen collection, geographic location of patient, and serotype results) and were included in the space–time scan analysis. Of these 34,032 cases, 4,544 (13%) were caused by serotype 1 IPD. Two clusters of serotype 1 were detected. The first (713 cases) occurred during May 2003–December 2004 and affected Gauteng Province and adjacent districts of Mpumalanga, Limpopo, and North-West Provinces (Figure 3, panel A; Technical Appendix Table 4). The second cluster (718 cases) occurred during September 2008–April 2012 and affected KwaZulu-Natal and Free State Provinces and adjacent districts of Gauteng, North-West, Mpumalanga, and Eastern Cape Provinces (Figure 3, panel B; Technical Appendix Table 4). We also assessed clustering of disease caused by 2 other epidemic-prone serotypes (serotypes 5 and 8); neither showed significant increases in case numbers compared with numbers in 2005.

## Discussion

In South Africa, serotype 1 pneumococcal disease had a number of distinct features. Children <5 years of age with serotype 1 IPD were less likely to die than were children with disease caused by other serotypes; this association between serotype 1 and death was not seen in older children and adults. Patients with serotype 1 IPD had fewer cases of penicillin-nonsusceptible disease, a lower prevalence of HIV, and less severe disease than patients with non–serotype 1 IPD. However, pneumonia and bacteremia occurred more commonly in patients with serotype 1 IPD than in patients with IPD caused by other serotypes.

Serotype 1 IPD incidence differed by geographic area and year, reflecting its epidemic potential (*1*). In older children and adults, serotype 1 was the most common serotype over the entire study period, even though numbers were lower after PCV13 introduction. Before PCV7 introduction, serotype 1 was the sixth most common serotype in children <5 years of age; by 2013, it no longer ranked in the top 10 serotypes in this age group.

 IPD is common in children with underlying diseases, including HIV. Compared with infections caused by other common pneumococcal serotypes, serotype 1 IPD was associated with more bacteremic pneumonia and peritonitis, occurred in older children and specific ethnic groups, and affected otherwise healthy children (*3*).

Serotype 1 IPD has marked temporal variability (*19*) and is associated with outbreaks (*20*,*21*). In our study, we noted fluctuations in incidence rates for serotype 1 IPD, especially among young children before PCV introduction. Incidence of serotype 1 IPD decreased in all age groups after 2011, likely due to the effect of PCV13, and serotype 1 disease nearly disappeared among the youngest children by 2013, two years after PCV13 introduction (*10*). We cannot exclude that other factors (e.g., improvements in access to antiretroviral treatment and programs for the prevention of mother-to-child HIV transmission) may have contributed to this decrease (*14*,*22*). We identified 2 large clusters that were not recognized prospectively because of the difficulty in identifying communitywide clusters in real time, especially using laboratory-based surveillance.

Our findings showed differences in the geographic distribution of serotype 1 and non–serotype 1 disease. Serotype 1 has been described to occur more frequently in underprivileged populations in developing countries (*19*); in our study, differences in specimen collection practices between provinces may have contributed to differences seen in disease distribution, as shown in other studies (*23*). Similar to findings by others (*24**,**25*), we found a difference in serotype distribution by age: serotype 1 IPD incidence was proportionally similar among older children and adults compared with that among children <1 year of age, whereas other serotypes predominated in the youngest age group and showed only a small peak in young adults. A number of factors may contribute to these age-associated differences (*25*). Compared with other serotypes, serotypes 1 and 5 are rarely carried by healthy persons; a short duration of carriage results in less opportunity for recombination events and less antibiotic selection pressure, resulting in reduced antibiotic nonsusceptibility in serotype 1 isolates (*26*).

Similar to findings in other studies (*3*), we found that, compared with other pneumococcal serotypes, serotype 1 caused more bacteremic pneumonia than meningitis. In addition, among HIV-uninfected children, serotype 1 IPD made up a larger proportion of disease than in HIV-infected children (*27*,*28*), suggesting that serotype 1 is more invasive and virulent, thus affecting otherwise healthy persons (*29*,*30*). Among children <5 years of age, those with serotype 1 disease were less likely to die than those with disease caused by other serotypes (*31*), and those most at risk of death were the very young (<1 year of age) and those HIV infected. In older persons, no association was found between serotype 1 disease and death when compared with other serotypes. Another analysis from the prevaccine era showed an increased risk of death among adults with serotype 1 disease compared with those with serotype 4 disease (*32*); this increased risk has been shown in few other studies (*33*).

Our study had several limitations. First, we included only patients who sought care at healthcare facilities with laboratories that submitted pneumococcal isolates to the National Institute for Communicable Diseases and who had specimens collected; patients with mild clinical pneumococcal disease treated in the community were not included. Second, we were able to map serotype 1 IPD incidence only at district level, so minor changes in incidence and clusters at the individual healthcare facility level may have been missed. Third, because of the small number of patients in the <5-year-old age group, we did not show clusters by age. We expect that reported clusters would have been similar for all ages. Fourth, we did not collect details regarding duration of symptoms before admission and thus could not assess whether intensity of symptoms when healthcare was sought affected case-fatality rates. Fifth, PCR serotype results from samples with a *lytA* cycle threshold (C_t_) of >35 may not be accurate. We did not use PCR results in the trend analysis, and the proportion of *lytA* samples with high C_t_ values was low in the surveillance program (*34*), so the C_t_ accuracy is unlikely to have affected our results. Sixth, we used non–serotype 1 cases as our comparison group in the descriptive factor analysis; although this group changed over the study period, PCV13 serotypes (excluding serotype 1) made up >50% of this group until 2012 and 40% in 2013. Last, our study covered only a short period of observation after PCV13 introduction, making it difficult to determine whether reductions in serotype 1 IPD were due to introduction of this vaccine.

In conclusion, compared with IPD caused by other serotypes, IPD caused by serotype 1 in South Africa was characterized by shorter hospital stays, fewer cases of resistant disease, fewer in-hospital fatalities in children <5 years of age, and lower prevalence among HIV-infected persons. Serotype 1 caused disease in all age groups, although prevalence peaked in older children and young adults. Temporal reductions in serotype 1 IPD have been observed within 2 years of PCV13 introduction in South Africa; this observation must be corroborated by ongoing surveillance over an extended period of time.

Technical Appendix Methods and case comparisons from enhanced and nonenhanced sites, South Africa, 2003–2013.
