# Supplementary material for: Epidemiology of Serotype 1 Invasive Pneumococcal Disease, South Africa, 2003–2013
Source: Emerg Infect Dis. 2016 Feb;22(2):261–70. doi: 10.3201/eid2202.150967 (PMC4734528; doi:10.3201/eid2202.150967)
Supplement: Technical Appendix — Methods and case comparisons from enhanced and nonenhanced sites, South Africa, 2003–2013. [file 15-0967-Techapp-s1.pdf]

Article DOI: http://dx.doi.org/10.3201/eid2202.150967

Epidemiology of Serotype 1 Invasive Pneumococcal Disease, South Africa, 2003–2013

Technical Appendix

Methods

Invasive Pneumococcal Disease Surveillance in South Africa

Invasive pneumococcal disease (IPD) surveillance began in South Africa in 1999 (*1*) and was limited to the collection of laboratory data and isolates from pneumococcal cases. The surveillance program was expanded in 2003 through GERMS-SA (Group for Enteric, Respiratory and Meningeal Disease Surveillance in South Africa), a national, active, laboratory-based surveillance system. The number of hospitals and laboratories covered by the surveillance increased over time, however more than 70% of hospitals remained consistent in the program over most of the reported period (*2*).

All laboratories record basic demographic information (age, sex, date of specimen collection, and source of isolate) for all pneumococcal isolates. Enhanced surveillance with trained surveillance officers at 24 sentinel hospitals located in all nine provinces of South Africa, includes the collection of additional clinical data, for example, admission and discharge date, HIV serologic status, vaccination information and discharge diagnosis and outcome. Enhanced surveillance sites account for ≈50% of all reported pneumococcal cases nationally.

Enhanced surveillance sites were chosen based on convenience, interest from site investigators and number of isolates submitted. Larger sites with higher isolate submissions were favored, resulting in enhanced sites being mainly tertiary and some secondary (regional) hospitals. Non-enhanced sites include district, regional and tertiary public hospitals, private hospitals and clinics. The regional and tertiary hospitals however made up over 70% of isolates sent from non-enhanced sites.

To identify missed unreported cases, annual laboratory audits were conducted throughout the study period using a centralized National Health Laboratory Service Corporate Data Warehouse which consolidates cases for all public-sector laboratories. Audit cases were included in the surveillance database for incidence rate calculations. Cases were likely missed as isolates were submitted by staff working in busy routine clinical microbiological laboratories. Isolates were often delayed at the sites and submitted in batches with other surveillance organisms sent to the NICD. As *S. pneumoniae* is fastidious it was often non-viable by the time it reached the NICD.

Definitions

At enhanced sites where additional clinical information was available, underlying conditions were defined as asplenia, including sickle cell anemia; chronic illness (chronic lung, renal, liver, cardiac disease and diabetes); other immunocompromising conditions (excluding HIV), including organ transplant and malignancy; and other risk factors, including head injury with possible CSF leak, neurologic disorders, burns, chromosomal abnormalities, alcohol use and smoking. Clinical diagnoses were based on documented discharge diagnoses in patient medical records, with clinical syndrome separated into three groups: meningitis, bacteremic pneumonia, and bacteremia without focus/other. Pitt bacteremia score was calculated using 5 parameters: (1) oral temperature, (2) hypotension, (3) receipt of mechanical ventilation, (4) cardiac arrest, and (5) mental status. Severe disease was defined as a score of ≥4 points (*3*).

Serotypes were defined as serotype 1 or non-serotype 1 IPD. Penicillin non-susceptibility was categorized using 2013 Clinical and Laboratory Standards Institute breakpoints for oral penicillin V (susceptible, ≤0.06 μg/L; intermediately resistant, 0.12–1 μg/L and resistant, ≥2 μg/L) (*4*). Intermediately resistant and resistant groups were combined into a non-susceptible group for analysis. Pneumococcal disease was considered recurrent if diagnosed >21 days after a previous case in the same patient.

Other Interventions Affecting Invasive Pneumococcal Disease Trends in South Africa

Comprehensive HIV/AIDS treatment programs were implemented in South Africa in 2003 and access to treatment improved steadily with 80% coverage reported by 2012 (*5*).

Prevention of mother-to-child transmission programs also improved steadily with an associated decrease in mother-to-child HIV transmission rates from 12% in 2007 to 2.7% in 2011 (*6*) and 2.5% during 2012/2013 (*7*). This was despite a relatively constant prevalence of HIV in pregnant women of around 30% over the same period.

A manuscript describing the reduction in IPD in South Africa following the introduction of PCV (*2*) showed a 49% reduction in all serotype IPD and 85% reduction in PCV7 serotypes in HIV-uninfected children <2 years of age by 2012. In HIV-infected children PCV7 serotypes decreased by 86% and non-vaccine serotypes by 31% which showed the benefit of improvements in prevention of mother-to-child transmission of HIV, antiretroviral treatment in children and PCV7. Reductions in PCV13-serotype disease in 2009 and 2010, before the introduction of PCV13, were also most likely a result of ART. In HIV-infected children it was thought to be difficult to tease out the exact amount of reduction in pneumococcal disease due to PCV and that due to other interventions.

References

1. Huebner RE, Klugman KP, Matai U, Eggers R, Hussey G. Laboratory surveillance for *Haemophilus influenzae* type B meningococcal, and pneumococcal disease. Haemophilus Surveillance Working Group. S Afr Med J. 1999;89:924–5. PubMed

2. von Gottberg A, de Gouveia L, Tempia S, Quan V, Meiring S, von Mollendorf C, et al. Effects of vaccination on invasive pneumococcal disease in South Africa. N Engl J Med. 2014;371:1889–99. PubMed http://dx.doi.org/10.1056/NEJMoa1401914

3. Paterson DL, Ko WC, Von Gottberg A, Mohapatra S, Casellas JM, Goossens H, et al. Antibiotic therapy for *Klebsiella pneumoniae* bacteremia: implications of production of extended-spectrum beta-lactamases. Clin Infect Dis. 2004;39:31–7. PubMed http://dx.doi.org/10.1086/420816

4. Clinical and Laboratory Standards Institute. Performance standards for antimicrobial susceptibility testing; twenty-third informational supplement. Document M100–S23.Wayne (PA): The Institute; 2013.

5. Johnson LF. Access to antiretroviral treatment in South Africa, 2004–2011. Southern African Journal of HIV Medicine. 2012;13:22–7.

6. Barron P, Pillay Y, Doherty T, Sherman G, Jackson D, Bhardwaj S, et al. Eliminating mother-to-child HIV transmission in South Africa. Bull World Health Organ. 2013;91:70–4. PubMed http://dx.doi.org/10.2471/BLT.12.106807

7. Massyn N, Day C, Dombo M, Barron P, English R, Padarath A. District health barometer 2012/13[cited 2014 Jul 31] . http://www.hst.org.za/publications/district-health-barometer-201213

Technical Appendix Table 1. Comparison of cases from GERMS-SA enhanced and non-enhanced sites for all age groups, 2003–2013

Variable	Enhanced sites n/N (%)	Non-enhanced sites n/N (%)	OR (95% CI)	p value
Age				<0.001
<1 y	3431/20,826 (16)	3470/23,397 (15)	1.21 (1.08–1.36)	
1–4 y	2899/20,826 (14)	2828/23,397 (12)	1.26 (1.12–1.41)	
5–9 y	1286/20,826 (6)	1587/23,397 (7)	0.99 (0.88–1.13)	
10–14 y	510/20,826 (2)	700/23,397 (3)	0.89 (0.77–1.04)	
15–24 y	1269/20,826 (6)	1551/23,397 (7)	1.00 (0.89–1.14)	
25–44 y	7909/20,826 (38)	9058/23,397 (39)	1.07 (0.96–1.19)	
45–64 y	2844/20,826 (14)	3371/23,397 (14)	1.04 (0.92–1.16)	
>64 y	678/20,826 (3)	832/23,397 (4)	Reference	
Sex				0.83
Female	10,686/20,984 (51)	12,510/24,516 (51)	Reference	
Male	10,298/20,984 (49)	12,006/24,516 (49)	1.00 (0.97–1.04)	
Province				<0.001
Gauteng	11,287/21,188 (53)	10,950/25,297 (43)	Reference	
Western Cape	3038/21,188 (14)	2864/25,297 (11)	1.03 (0.97–1.09)	
KwaZulu-Natal	3045/21,188 (14)	2321/25,297 (9)	1.27 (1.20–1.35)	
Eastern Cape	570/21,188 (3)	3101/25,297 (12)	0.18 (0.16–0.20)	
Free State	1365/21,188 (6)	1687/25,297 (7)	0.78 (0.73–0.85)	
Mpumalanga	726/21,188 (3)	1873/25,297 (7)	0.38 (0.34–0.41)	
North West	338/21,188 (2)	1510/25,297 (6)	0.22 (0.19–0.25)	
Limpopo	363/21,188 (2)	719/25,297 (3)	0.49 (0.43–0.56)	
Northern Cape	456/21,188 (2)	272/25,297 (1)	1.63 (1.40–1.89)	
Year				<0.001
2003	1927/21,188 (9)	1962/25,297 (8)	Reference	
2004	2297/21,188 (11)	2245/25,297 (9)	1.04 (0.96–1.13)	
2005	2488/21,188 (12)	2398/25,297 (9)	1.06 (0.97–1.15)	
2006	2202/21,188 (10)	2534/25,297 (10)	0.88 (0.81–0.96)	
2007	2148/21,188 (10)	2595/25,297 (10)	0.84 (0.77–0.92)	
2008	2051/21,188 (10)	2784/25,297 (11)	0.75 (0.69–0.82)	
2009	2039/21,188 (10)	2725/25,297 (11)	0.76 (0.70–0.83)	
2010	1918/21,188 (9)	2280/25,297 (9)	0.86 (0.78–0.93)	
2011	1615/21,188 (8)	2189/25,297 (9)	0.75 (0.69–0.82)	
2012	1305/21,188 (6)	1917/25,297 (8)	0.69 (0.63–0.76)	
2013	1198/21,188 (6)	1668/25,297 (7)	0.73 (0.66–0.81)	
Penicillin non-susceptibility				<0.001
Susceptible	10,536/16,338 (64)	10,986/16,510 (67)	Reference	
Non-susceptible	5802/16,338 (36)	5524/16,510 (33)	1.10 (1.05–1.15)	
Specimen type				<0.001
CSF	5697/21,188 (27)	11,446/25,297 (45)	Reference	
Blood culture	13,897/21,188 (66)	11,104/25,297 (44)	2.51 (2.41–2.62)	
Other specimens	1594/21,188 (8)	2747/25,297 (11)	1.17 (1.09–1.25)	
Serotype				<0.001
Non-serotype 1	19,246/21,186 (91)	22,690/25,294 (90)	Reference	
Serotype 1	1940/21,186 (9)	2604/25,294 (10)	0.88 (0.83–0.93)	

Technical Appendix Table 2. Characteristics of 10,899 patients ≥5 years of age with invasive pneumococcal disease caused by serotype 1 and non–serotype 1 *Streptococcus pneumoniae*, South Africa, 2003–2013*

	No. cases/no. total (%)		Univariate analysis†		Multivariable analysis†	
Variable	Serotype 1	Non–serotype 1	OR (95% CI)	p value	aOR (95% CI)	p value
Age group, y						
5–9	254/1,642 (15)	809/9,257 (9)	3.19 (2.29–4.45)	<0.001	13.48 (5.53–32.82)	<0.001
10–14	115/1,642 (7)	298/9,257 (3)	3.92 (2.71–5.66)		8.02 (3.15–20.43)	
15–24	201/1,642 (12)	755/9,257 (8)	2.71 (1.93–3.79)		5.65 (2.31–13.82)	
25–44	768/1,642 (47)	5,078/9,257 (55)	1.54 (1.13–2.10)		3.67 (1.53–8.76)	
45–64	257/1,642 (16)	1,839/9,257 (20)	1.42 (1.03–1.97)		2.57 (1.06–6.23)	
>64	47/1,642 (3)	478/9,257 (5)	Reference		Reference	
Black race	1452/1,576 (92)	7,854/8,889 (88)	1.54 (1.27–1.87)	<0.001		
Province						
Gauteng	951/1,642 (58)	4,804/9,257 (52)	Reference	<0.001	Reference	<0.001
Western Cape	99/1,642 (6)	1,443/9,257 (16)	0.35 (0.28–0.43)		0.24 (0.17–0.34)	
KwaZulu-Natal	228/1,642 (14)	1,469/9,257 (16)	0.78 (0.67–0.92)		0.80 (0.60–1.07)	
Eastern Cape	47/1,642 (3)	166/9,257 (2)	1.43 (1.03–1.99)		0.80 (0.39–1.63)	
Free State	130/1,642 (8)	516/9,257 (6)	1.27 (1.04–1.56)		0.89 (0.64–1.22)	
Mpumalanga	64/1,642 (4)	358/9,257 (4)	0.90 (0.69–1.19)		0.80 (0.43–1.49)	
North-West	34/1,642 (2)	148/9,257 (2)	1.16 (0.79–1.70)		2.25 (1.13–4.48)	
Limpopo	42/1,642 (3)	143/9,257 (2)	1.48 (1.04–2.11)		0.97 (0.47–2.01)	
Northern Cape	47/1,642 (3)	210/9,257 (2)	1.13 (0.82–1.56)		1.39 (0.85–2.26)	
Year of specimen collection						
2003	209/1,642 (13)	733/9,257 (8)	1.45 (1.16–1.80)	<0.001	1.17 (0.76–1.82)	0.01
2004	225/1,642 (14)	891/9,257 (10)	1.28 (1.03–1.58)		1.32 (0.87–2.00)	
2005	196/1,642 (12)	994/9,257 (11)	Reference		Reference	
2006	142/1,642 (9)	962/9,257 (10)	0.75 (0.59–0.95)		0.67 (0.42–1.09)	
2007	112/1,642 (7)	892/9,257 (10)	0.64 (0.50–0.82)		0.71 (0.44–1.14)	
2008	116/1,642 (7)	842/9,257 (9)	0.70 (0.55–0.89)		0.86 (0.56–1.32)	
2009	156/1,642 (10)	866/9,257 (9)	0.91 (0.73–1.15)		1.21 (0.80–1.84)	
2010	164/1,642 (10)	995/9,257 (11)	0.84 (0.67–1.05)		1.02 (0.66–1.57)	
2011	134/1,642 (8)	819/9,257 (9)	0.83 (0.65–1.05)		0.98 (0.63–1.51)	
2012	112/1,642 (7)	676/9,257 (7)	0.84 (0.65–1.08)		0.96 (0.62–1.48)	
2013	76/1,642 (5)	587/9,257 (6)	0.66 (0.49–0.87)		0.64 (0.40–1.04)	
Medical conditions/treatment						
Length of hospital stay, d						
≤3	481/1,443 (33)	2518/8,311 (30)	Reference	0.001	Reference	0.01
4–14	758/1,443 (53)	4289/8,311 (52)	0.93 (0.82–1.05)		0.86 (0.68–1.09)	
≥15	204/1,443 (14)	1504/8,311 (18)	0.71 (0.60–0.85)		0.64 (0.48–0.86)	
Previous hospital admission	166/1,153 (14)	2000/6,816 (29)	0.40 (0.34–0.48)	<0.001	0.45 (0.35–0.57)	<0.001
Underlying medical condition‡	310/953 (33)	2571/6,083 (42)	0.66 (0.57–0.76)	<0.001		
Antimicrobial drug use in previous 2 mo§	32/962 (3)	412/5,550 (7)	0.43 (0.30–0.62)	<0.001		
HIV infected	717/1,007 (71)	5373/6,338 (85)	0.44 (0.38–0.52)	<0.001	0.39 (0.31–0.49)	<0.001
Treated for TB in previous 3 mo	146/1,126 (13)	1373/6,659 (21)	0.57 (0.48–0.69)	<0.001	0.73 (0.57–0.95)	0.02
Died during hospitalization	461/1,422 (32)	2650/8,228 (32)	1.01 (0.90–1.14)	0.88		
Pneumococcal isolate characteristics						
Penicillin nonsusceptible¶	15/1,555 (1)	2916/8,829 (33)	0.02 (0.01–0.03)	<0.001	0.02 (0.01–0.04)	<0.001
Previous invasive pneumococcal disease**	26/1,642 (2)	396/9,257 (4)	0.36 (0.24–0.54)	<0.001	0.32 (0.16–0.63)	0.001
Clinical syndrome/specimen type						
Specimen type						
Cerebral spinal fluid	512/1,642 (31)	2626/9,257 (28)	Reference	0.05		
Blood	1025/1,642 (62)	5967/9,257 (64)	0.88 (0.78–0.99)			
Other	105/1,642 (6)	664/9,257 (7)	0.81 (0.65–1.02)			
Clinical syndrome††						
Meningitis	587/1,541 (38)	3043/8,793 (35)	Reference	0.02	Reference	0.006
Pneumonia	832/1,541 (54)	5076/8,793 (58)	0.85 (0.76–0.95)		1.28 (1.03–1.58)	
Bacteremia	122/1,541 (8)	674/8,793 (8)	0.94 (0.76–1.16)		1.76 (1.22–2.55)	

*All patients were reported from the enhanced Group for Enteric, Respiratory, and Meningeal Disease Surveillance in South Africa (GERMS-SA) surveillance sites. aOR, adjusted odds ratio; OR, odds ratio; TB, tuberculosis
†Only variables significant on univariate and multivariable analysis are shown (exception is death during hospital admission). Variables not included in table are sex, Pitt bacteremia score, antimicrobial drug in previous 24 h, and viable culture. Prematurity and malnutrition were not included in the analysis because they were not considered relevant or actively collected for patients ≥5 years of age.
‡Includes asplenia or sickle cell anemia; chronic illness (i.e., chronic lung, renal, liver, cardiac disease, and diabetes); other immunocompromising conditions (i.e., organ transplant, primary immunodeficiency, immunotherapy, and malignancy, but excluding HIV); and other risk factors (i.e., head injury with possible cerebral spinal fluid leak, neurologic disorders, burns, chromosomal abnormalities, smoking, and alcohol use).
§Use of any antimicrobial drug in 2 mo before admission.
¶Considered penicillin nonsusceptible at MIC ≥0.12 μg/mL; intermediately resistant and resistant groups were combined into a nonsusceptible group.
**Invasive pneumococcal disease diagnosis >21 days before this episode.
††Clinical diagnoses were made on the basis of documented discharge diagnoses in patient medical records; clinical syndrome separated into 3 groups: meningitis, bacteremic pneumonia, and bacteremia without focus or other diagnosis (e.g., septic arthritis, endopthalmitis, peritonitis, pericarditis).

Technical Appendix Table 3. Factors associated with death in patients ≥5 years of age with serotype 1 invasive pneumococcal disease, South Africa, 2003–2013*

	Univariate analysis			Multivariable analysis	
Variable	No. deaths/no. cases (%)	OR (95% CI)	p value	aOR (95% CI)	p value
Demographic/socioeconomic characteristic					
Age group, y					
5–9	37/350 (11)	Reference	<0.001	Reference	<0.001
10–14	23/143 (16)	1.62 (0.92–2.84)		1.24 (0.65–4.57)	
15–24	90/362 (25)	2.80 (1.85–4.24)		3.05 (1.47–6.32)	
25–44	611/1,950 (31)	3.86 (2.71–5.50)		5.07 (2.74–9.38)	
45–64	285/686 (42)	6.01 (4.14–8.73)		9.00 (4.66–17.35)	
>64	58/133 (44)	6.54 (4.03–10.61)		10.13 (4.46–23.00)	
Race					
Nonblack	61/250 (24)	Reference	0.03		
Black	1,023/3,313 (31)	1.38 (1.02–1.86)			
Province					
Gauteng	706/2,444 (29)	Reference	<0.001		
Western Cape	54/217 (25)	0.82 (0.59–1.12)			
KwaZulu-Natal	98/329 (30)	1.04 (0.81–1.34)			
Eastern Cape	29/68 (43)	1.83 (1.12–2.98)			
Free State	59/189 (31)	1.12 (0.81–1.54)			
Mpumalanga	63/154 (41)	1.70 (1.22–2.38)			
North-West	34/70 (49)	2.32 (1.44–3.75)			
Limpopo	42/94 (45)	1.99 (1.31–3.01)			
Northern Cape	19/59 (32)	1.17 (0.67–2.03)			
Medical condition/treatment					
Length of hospital stay, d					
≥3	750/1,130 (66)	Reference	<0.001	Reference	<0.001
4–14	254/1,891 (13)	0.08 (0.07–0.09)		0.07 (0.05–0.10)	
≥15	93/577 (16)	0.10 (0.08–0.13)		0.06 (0.04–0.09)	
Pitt bacteremia score†					
0–3	744/2,920 (26)	Reference	<0.001	Reference	<0.001
≥4	258/361 (71)	7.33 (5.74–9.34)		5.26 (3.53–7.84)	
Underlying medical condition‡					
No	357/1,582 (23)	Reference	<0.001	Reference	0.004
Yes	257/827 (31)	1.55 (1.28–1.87)		1.53 (1.14–2.04)	
Antimicrobial drug use in 24 h before admission					
No	644/2,537 (25)	Reference	0.05		
Yes	32/93 (34)	1.54 (1.00–2.39)			
HIV status					
HIV uninfected	108/514 (21)	Reference	0.001		
HIV infected	610/2,165 (28)	1.47 (1.17–1.86)			
Treated for tuberculosis in previous 3 mo					
No	508/2,156 (24)	Reference	0.001	Reference	0.001
Yes	154/496 (31)	1.46 (1.18–1.81)		1.75 (1.25–2.45)	
Previous invasive pneumococcal disease§					
No	1097/3,536 (31)	Reference	<0.001		
Yes	7/88 (8)	0.19 (0.09–0.42)			
Clinical syndrome/specimen type					

Specimen type					
Cerebral spinal fluid	461/802 (57)	Reference	<0.001		
Blood	565/2,440 (23)	0.22 (0.19–0.26)			
Other	78/382 (20)	0.19 (0.14–0.25)			
Clinical syndrome¶					
Meningitis	531/982 (54)	Reference	<0.001	Reference	<0.001
Pneumonia	490/2,311 (21)	0.23 (0.19–0.27)		0.18 (0.13–0.25)	
Bacteremia	75/307 (24)	0.27 (0.21–0.37)		0.29 0.18–0.48)	

*All patients were reported from the enhanced Group for Enteric, Respiratory, and Meningeal Disease Surveillance in South Africa (GERMS-SA) surveillance sites. Only variables significant on univariate and multivariable analysis are shown. Variables not included in table are sex, year, previous hospital admission, any antimicrobial drug used in 2 mo before admission, and penicillin-nonsusceptible invasive pneumococcal disease. Prematurity and malnutrition were not included in the analysis because they were not considered relevant or actively collected for patients ≥5 years of age. aOR, adjusted odds ratio; OR, odds ratio.

†Pitt bacteremia score calculated by using temperature, hypotension, mechanical ventilation, cardiac arrest, and mental status. Severe disease defined as score of ≥4 points.

‡Includes asplenia or sickle cell anemia; chronic illness (i.e., chronic lung, renal, liver, cardiac disease and diabetes); other immunocompromising conditions (i.e., organ transplant, primary immunodeficiency, immunotherapy, and malignancy, but excluding HIV); and other risk factors (i.e. head injury with possible cerebral spinal fluid leak, neurologic disorders, burns, and chromosomal abnormalities).

§Invasive pneumococcal disease diagnosis >21 days before this episode.

¶Clinical diagnoses were made on the basis of documented discharge diagnoses in patient medical records, with clinical syndrome separated into 3 groups: meningitis, bacteremic pneumonia, and bacteremia without focus or other diagnosis (e.g. septic arthritis, endopthalmitis, peritonitis, pericarditis).

Technical Appendix Table 4. Serotype 1 clusters, by district, in South Africa, 2003–2013

		Location			
Cluster	Period	District	Province	Relative risk	p value
1	May 2003–Dec 2004	City of Johannesburg	Gauteng	1.7	<0.001
		City of Tshwane	Gauteng		
		Ekurhuleni	Gauteng		
		Metweding	Gauteng		
		Sedibeng	Gauteng		
		West Rand	Gauteng		
		Sekhukhune Cross	Limpopo		
		Govan Mbeki	Mpumalanga		
		Nkangala	Mpumalanga		
		Bojanala	North-West		
		Southern	North-West		
2	Sep 2008–Apr 2012	Alfred Nzo	Eastern Cape	1.4	<0.001
		Amatole	Eastern Cape		
		Chris Hani	Eastern Cape		
		Ukhahlamba	Eastern Cape		
		Lejweleputswa	Free State		
		Motheo	Free State		
		Northern	Free State		
		Thabo Mofutsanyane	Free State		
		Xhariep	Free State		
		Ekurhuleni	Gauteng		
		Sedibeng	Gauteng		
		Amabuja	KwaZulu-Natal		
		Ethekwini	KwaZulu-Natal		
		iLembe	KwaZulu-Natal		
		Sisonke	KwaZulu-Natal		
		Ugu	KwaZulu-Natal		
		UMgungundlovu	KwaZulu-Natal		
		Umkhanyakude	KwaZulu-Natal		
		Umzinyathi	KwaZulu-Natal		
		Uthukela	KwaZulu-Natal		
		Uthungulu	KwaZulu-Natal		
		Zululand	KwaZulu-Natal		
		Govan Mbeki	Mpumalanga		
		Southern	North-West		

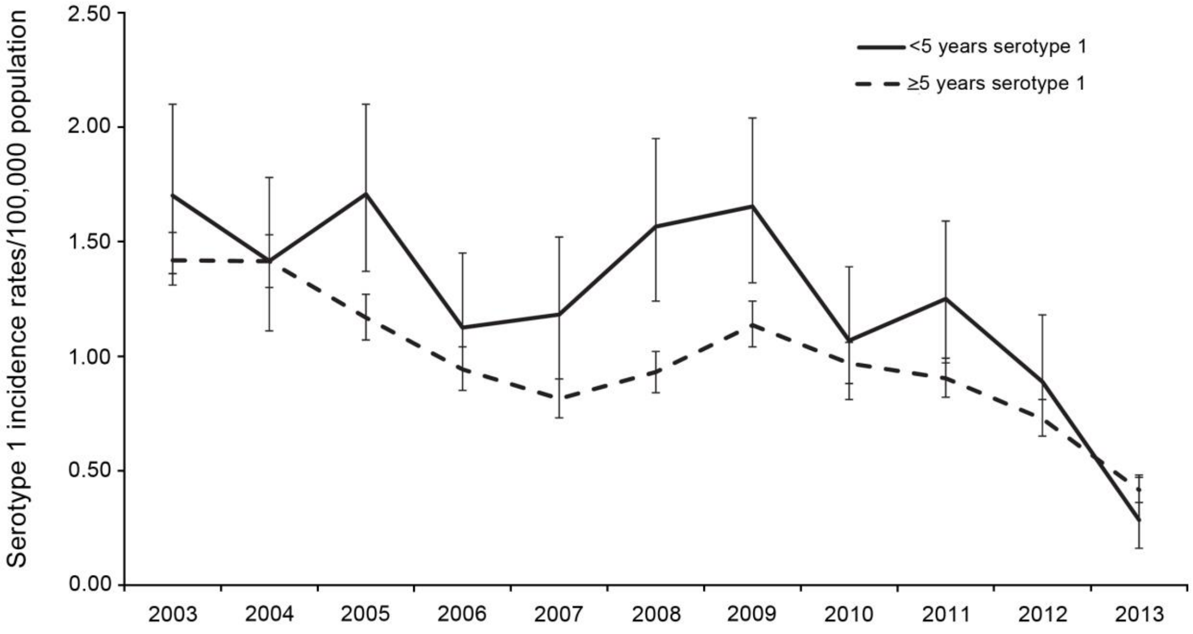

Technical Appendix Figure. Incidence rates for serotype 1 in children <5 years (n = 714) and individuals ≥5 years (n = 5167) of age, South Africa, 2003–2013. Error bars indicate CIs for incidence rates. N, imputed serotype 1 cases.
